# Incidence, Risk Factors, and Outcomes of Periprosthetic Fractures Following Total Hip and Knee Arthroplasty: A Multicenter Retrospective Study

**DOI:** 10.7759/cureus.99611

**Published:** 2025-12-19

**Authors:** Shaiza Shoaib, Maria Mir, Ruba Irshad, Ruquiya Shaikh, Madiha Imtiaz, Qurat Ul Ain Khan, Zain Ramzan

**Affiliations:** 1 Department of Orthopaedic Surgery, Combined Military Hospital (CMH) Kharian Medical College, Kharian, PAK; 2 Department of Neurosurgery, Brain Surgery Hospital, Rawalpindi, PAK; 3 Department of Biochemistry, Army Medical College, National University of Medical Sciences (NUMS), Rawalpindi, PAK; 4 Department of Physical Therapy, School of Physiotherapy, Karachi Metropolitan University, Karachi, PAK; 5 Department of Radiology, Islamabad Diagnostic Center, Islamabad, PAK; 6 Department of Anatomy, Liaquat College of Medicine and Dentistry, Karachi, PAK; 7 Department of Medicine, Amna Inayat Medical College, Lahore, PAK

**Keywords:** arthroplasty, hip, incidence, knee, periprosthetic fractures, replacement, risk factors, treatment outcome

## Abstract

Introduction: Total hip arthroplasty (THA) and total knee arthroplasty (TKA) are effective procedures for pain relief and functional restoration, but periprosthetic fractures (PPFs) remain a serious complication, associated with functional decline, complex management, and higher healthcare burden.

Objective: To determine the incidence, risk factors, and outcomes of PPF following THA and TKA in a multicenter cohort.

Methodology: A retrospective study was conducted across three tertiary hospitals in Pakistan, including 327 arthroplasty procedures from January 2022 to December 2023. Demographic, clinical, surgical, and follow-up data were reviewed. The primary outcome was the incidence of PPF, while secondary outcomes included risk factors and postoperative complications. Statistical analyses were performed using chi-square, Fisher’s exact, t-test, or Mann-Whitney U test, with logistic regression applied to identify independent predictors. Model fit was assessed with the Hosmer-Lemeshow test, using SPSS version 26.0 (IBM Corp., Armonk, NY), with significance set at p < 0.05.

Results: PPF occurred in 15 patients (4.6%), more frequently after THA (10, 5.8%) than TKA (5, 3.2%). Independent risk factors included age ≥70 years (adjusted odds ratio (aOR): 2.56, 95% CI: 1.04-6.29), osteoporosis (aOR: 3.14, 95% CI: 1.21-8.15), and revision surgery (aOR: 2.89, 95% CI: 1.03-8.10). Treatments included open reduction and internal fixation (ORIF) (11, 73.3%) and revision arthroplasty (4, 26.7%). Complications occurred in five patients (33.3%), including re-operation in three (20%) and mortality in one (6.7%).

Conclusion: PPFs after arthroplasty are strongly linked to older age, osteoporosis, and revision surgery. Optimizing bone health and careful surgical planning are crucial to reduce risk and improve outcomes.

## Introduction

Patients with advanced osteoarthritis and other degenerative joint diseases can significantly improve their quality of life, joint function, and pain relief with total hip arthroplasty (THA) and total knee arthroplasty (TKA), two of the most popular and successful orthopedic procedures performed globally [1]. The need for arthroplasty is growing worldwide because of advancements in perioperative care, prosthetic design, and surgical procedures, especially as the population ages and life expectancy rises [2]. However, as the use has increased, there have also been related consequences, with periprosthetic fractures (PPFs) being one of the most difficult to manage [3].

PPFs are defined as fractures occurring in the bone surrounding an implanted prosthesis. They represent a significant cause of morbidity following THA and TKA and pose unique challenges in terms of diagnosis, surgical management, and postoperative recovery [4]. These fractures not only threaten the stability and survival of the implant but also frequently require complex revision surgery, extended hospital stays, and prolonged rehabilitation [5]. Consequently, PPFs contribute to higher healthcare costs and increased patient burden, making them an important area of clinical concern [6].

The reported incidence of PPFs has been steadily increasing over the past two decades [7]. This rise can be attributed to the growing number of primary arthroplasties being performed, the aging population with fragile bone quality, and the extended survivorship of prostheses, which increases the cumulative lifetime risk of fracture [8]. Studies estimate that the incidence of PPFs following THA ranges between 0.1% and 4%, while for TKA, rates are reported between 0.3% and 2.5%, though these figures vary depending on population characteristics and study design [9]. Importantly, as implant longevity improves, fractures around both primary and revision prostheses are expected to become more frequent [10].

Many risk factors for PPFs have been recognized, including advanced age, female gender, osteoporosis, rheumatoid arthritis, chronic corticosteroid use, previous revision arthroplasty, and technical aspects such as implant malalignment or loosening. Chronic steroid use refers to prolonged systemic corticosteroid therapy, typically ≥5 mg/day of prednisone (or equivalent) for ≥3 months. Such exposure reduces bone mineral density and impairs bone remodeling, leading to secondary osteoporosis and increased susceptibility to PPFs [11]. Additionally, high-energy trauma, low bone mineral density, and perioperative complications contribute to the likelihood of sustaining a fracture [12]. Understanding these risk factors is essential for promptly identifying those who are at risk and putting preventative measures into action [13].

The outcomes of PPFs are often unfavorable compared to other complications of arthroplasty. Patients with PPFs are at higher risk for functional decline, impaired mobility, infection, and even mortality [14]. Depending on the type of fracture, implant stability, and bone stock, surgical management is often complicated and calls for specific procedures such as revision arthroplasty, open reduction and internal fixation (ORIF), or a combination of both [15]. Despite advances in surgical management, complication rates remain high, with significant risks of nonunion, implant failure, and recurrent fracture [16]. Consequently, PPFs present an important issue for patients and healthcare systems, highlighting the necessity of more research into how to prevent and treat them [17].

Despite increasing recognition of PPFs as a growing clinical problem, much of the existing literature originates from single-center experiences or registry-based data, often with limited focus on combined outcomes of both hip and knee arthroplasty-related fractures [18]. Comprehensive multicenter analyses exploring the incidence, risk factors, and outcomes in diverse patient populations remain relatively scarce [19].

Therefore, this study aims to evaluate the incidence, risk factors, and outcomes of PPFs following total hip and knee arthroplasty through a multicenter retrospective analysis, thereby addressing a key gap in the current literature.

## Materials and methods

A multicenter retrospective study was conducted across three tertiary care hospitals, Lady Reading Hospital (LRH), Khyber Teaching Hospital (KTH), and Hayatabad Medical Complex (HMC), in Peshawar, Pakistan, over a two-year period. The study reviewed medical records of patients who underwent primary or revision THA or TKA between 1 January 2022 and 31 December 2023. Medical records of all adult patients (≥18 years) who had primary or revision THA or TKA during the study period were screened. Patients were included if they had adequate clinical and radiographic documentation available in the hospital record system and were followed at least until discharge or until a PPF was documented. Patients were excluded if records were irretrievable, key data were missing (implant type, operative date, or outcome), or if the procedure was performed for oncologic resection or arthroplasty. Additionally, patients with documented pathological fractures secondary to malignancy, known metabolic bone diseases (such as hyperparathyroidism), severe hypocalcemia, or untreated severe vitamin D deficiency were excluded when these conditions were clearly identified in the medical records. Functional outcomes (such as postoperative mobility, ability to ambulate, and documented return to baseline activity) were recorded when available from physiotherapy and follow-up notes to enhance clinical interpretation of results. The standard formula for estimating a single proportion (n = *z*^2^* *p* (1-*p*)/*d*^2^) was used to get the necessary sample size [20]. Where *d* is the desired absolute precision, *p* is the estimated incidence of PPF, and *Z* is the Z-score at a 95% confidence level (1.96). Based on prior literature, an expected incidence (p) of 3.5% (0.035) [21] and a precision (d) of 2.0% (0.02) were used. The calculated sample size was 324.38, which was rounded up to 325. To account for potential incomplete or unusable records in this retrospective review, the final targeted sample size was set at 327 patient procedures.

Data collection

Trained research assistants extracted data from electronic medical records and paper charts using a standardized data extraction form that had been pilot tested on 20 records. Collected variables included demographic data (age, sex, BMI), comorbidities (diabetes, vitamin D deficiency, serum calcium levels, osteoporosis, steroid use), history of malignancy, hyperparathyroidism, and other metabolic abnormalities (such as hypocalcemia), indication for arthroplasty (osteoarthritis, trauma, avascular necrosis, revision), operative details (primary vs. revision, implant type, fixation method), perioperative variables (operative time, blood loss), and follow-up outcomes (occurrence and timing of PPF, fracture classification, management, in-hospital complications, length of stay, and mortality). Radiographs and operative notes were reviewed to confirm fracture diagnosis and to classify PPFs according to established systems (e.g., Vancouver for hip and appropriate classification for knee fractures). Serum vitamin D and calcium levels measured within three months before surgery were documented, where available, to assess their potential confounding influence on bone fragility. Bone turnover markers (BTMs), which are protein or protein-derivative biomarkers released during bone remodeling by osteoblasts and osteoclasts, were not routinely assessed in the participating centers; therefore, these biomarkers were not included in the analysis. Additionally, serum vitamin D and calcium levels were not consistently available for all patients, and their assessment was limited. The incidence of PPF after THA or TKA over the study period was the primary outcome. Secondary outcomes included clinical outcomes following fracture (treatment modality, complications, re-operation, and mortality) and factors linked to PPF (risk factors connected to patients, surgeries, and implants). Functional outcomes, including postoperative ambulation status and documented return to mobility, were evaluated when available to supplement clinical outcome assessment. Each chart was reviewed by one data extractor, and a random 10% sample was rechecked by a second reviewer to ensure accuracy and inter-rater reliability. Discrepancies were resolved by consensus and by consulting the operating surgeon’s record when needed. Data were entered into a secure, password-protected database with de-identified study IDs. Missing data were documented; records with critical missing variables (as defined in the eligibility criteria) were not included in the analysis.

Statistical analysis

IBM SPSS version 26 (IBM Corp., Armonk, NY) was used to analyze the data. Categorical variables were displayed as counts and percentages, while continuous variables were summarized as mean ± standard deviation or median with interquartile range based on distribution. The number of fractures divided by the total number of arthroplasty surgeries covered was used to determine the incidence of PPFs. The t-test or Mann-Whitney U test, when applicable, and the chi-square or Fisher's exact test, for categorical factors, were employed in univariate analyses to investigate associations with PPF. To account for potential confounding variables, clinically significant covariates and variables with p < 0.10 on univariate testing were added to a multivariable logistic regression model. Serum vitamin D and calcium levels were included as covariates in the regression model when available to assess potential confounding effects. The Hosmer-Lemeshow goodness-of-fit test was used to evaluate model fitness, and adjusted odds ratios (aOR) with 95% CIs were presented [22]. Statistical significance was established at a two-sided p-value of less than 0.05.

## Results

**Figure 1:**
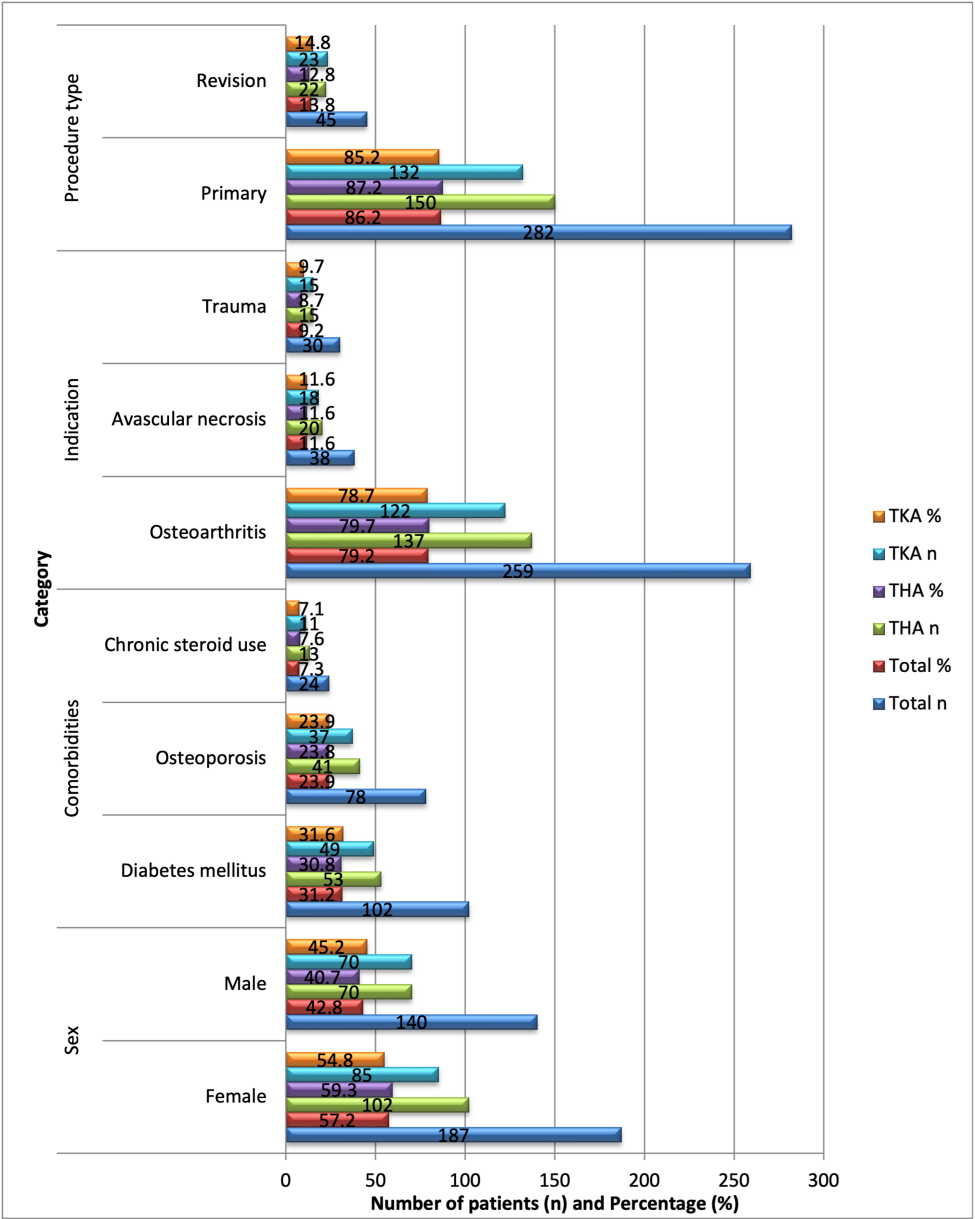
Baseline characteristics of the study population (n = 327). Statistical significance was assessed using chi-square or t-test as appropriate. No significant differences were observed between groups (p > 0.05). THA: total hip arthroplasty; TKA: total knee arthroplasty.

A total of 327 patient procedures were included in the study, and their mean age was 67.8 ± 9.5 years. There were 140 (42.8%) males and 187 (57.2%) females in the group. The mean body mass index (BMI) was 28.4 ± 4.1 kg/m². Common comorbidities included diabetes mellitus in 102 (31.2%), osteoporosis in 78 (23.9%), and chronic steroid use in 24 (7.3%) patients, as illustrated in Figure 1. The majority of procedures were primary arthroplasties, representing 282 (86.2%) of cases, while revisions accounted for 45 (13.8%). Osteoarthritis was the most frequent indication for surgery, observed in 259 (79.2%) patients, followed by avascular necrosis in 38 (11.6%) and trauma-related indications in 30 (9.2%). Statistical analysis using chi-square tests indicated that comorbidities were evenly distributed between the THA and TKA groups (p > 0.05), suggesting a comparable baseline clinical profile across both procedure types (Figure 1).

During the two-year study period, 15 patients (4.6%) developed PPFs following arthroplasty. Fractures were more frequent after THA, with 10 cases (5.8%), compared to five cases (3.2%) following TKA, highlighting a higher incidence of PPF in hips compared to knees. The median time to fracture was 8.2 months (interquartile range: 3-14 months). As shown in Table 1, hip fractures were classified using the Vancouver system: B1 in four patients (40%), B2 in three patients (30%), and C in three patients (30%). Knee fractures were classified as Su type I in three patients (60%) and Su type II in two patients (40%). Fisher's exact test did not reveal a statistically significant difference in the incidence of fractures between THA and TKA (p = 0.271) (Table 1).

**Table 1:** Incidence and type of periprosthetic fractures. Incidence represents the proportion of patients who developed periprosthetic fractures. Fisher’s exact test was used to compare fracture incidence between THA and TKA. P > 0.05, not statistically significant. THA: total hip arthroplasty; TKA: total knee arthroplasty.

Procedure	Fractures	Incidence (%)	Fracture type	Statistical test	p-value
THA	10	5.8	Vancouver B1: 4	Fisher’s exact	0.271
			Vancouver B2: 3. Vancouver C: 3		
TKA	5	3.2	Su I: 3	Fisher’s exact	0.271
			Su II: 2		
Total	15	4.6			

A number of variables, including age ≥70 years, osteoporosis, revision surgeries, and female gender, were linked to an elevated incidence of PPFs by univariate analysis. Chi-square tests showed significant associations for age ≥70 years (7/15, 46.7% vs. 93/312, 29.8%; p = 0.032), osteoporosis (9/15, 60% vs. 69/312, 22.1%; p = 0.014), and revision procedures (5/15, 33.3% vs. 40/312, 12.8%; p = 0.041). As shown in Table 2, female gender demonstrated a trend toward significance (10/15, 66.7% vs. 177/312, 56.7%; p = 0.078). Multivariable logistic regression verified that age ≥70 years (adjusted odds ratio (aOR): 2.56, 95% CI: 1.04-6.29; p = 0.041), osteoporosis (aOR: 3.14, 95% CI: 1.21-8.15; p = 0.018), and revision procedures (aOR: 2.89, 95% CI: 1.03-8.10; p = 0.044) were independent risk factors for PPF. The multivariable model did not show statistical significance for female gender (aOR: 1.82, 95% CI: 0.69-4.83; p = 0.231) (Table 2).

**Table 2:** Multivariable logistic regression for risk factors of PPF. Logistic regression was used to identify independent risk factors for periprosthetic fractures. Model fit was assessed using the Hosmer–Lemeshow goodness-of-fit test (χ² = 6.52, p = 0.59). Statistical significance was defined as p < 0.05. aOR = adjusted odds ratio; CI = confidence interval.

Risk factor	aOR (95% CI)	p-value
Age ≥70 years	2.56 (1.04–6.29)	0.041
Female gender	1.82 (0.69–4.83)	0.231
Osteoporosis	3.14 (1.21–8.15)	0.018
Revision procedure	2.89 (1.03–8.10)	0.044

**Figure 2:**
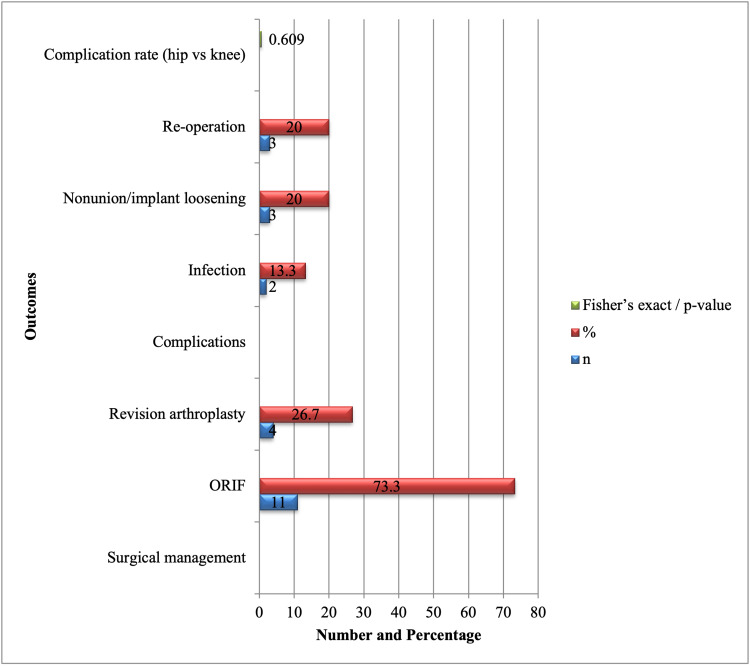
Management and post-fracture outcomes (n = 15). Data are presented as numbers (n) and percentages (%) or median (IQR) where appropriate. Fisher’s exact test was applied to compare complication rates between hip and knee fractures, showing no statistically significant difference (p = 0.609). Statistical significance was defined as p < 0.05. ORIF: open reduction and internal fixation.

All 15 patients who sustained PPFs were managed surgically. Six patients (60%) underwent ORIF for hip fractures, while four patients (40%) underwent revision arthroplasty, as shown in Figure 2. All knee fractures were treated with ORIF (5, 100%). Postoperative complications occurred in five patients, including infections in two cases (13.3%) and nonunion or implant loosening in three cases (20.0%). Re-operation was necessary in three patients (20.0%), most commonly due to mechanical failure. The median hospital stay was 12 days (IQR: 9-17), reflecting the significant clinical and resource burden of these fractures. One patient (6.7%) died within 30 days postoperatively. Fisher's exact test revealed that there was no statistically significant difference in the complication rates between the groups with hip and knee fractures (p = 0.609), indicating similar postoperative results for both operations (Figure 2).

Patients who developed PPFs demonstrated significantly greater intraoperative burden compared to those without fractures. As shown in Table 3, the median operative time was longer in the PPF group at 145 minutes (IQR: 130-165) compared to 120 minutes (IQR: 100-135) in the non-PPF group, with the difference reaching statistical significance (p = 0.026, Mann-Whitney U test). Similarly, intraoperative blood loss was higher among PPF patients, with a mean of 480 ± 120 mL compared to 360 ± 90 mL in those without fractures, which was also statistically significant (p = 0.011, t-test) (Table 3).

**Table 3:** Perioperative variables in patients with and without PPF. Data are presented as mean ± SD or median (IQR). Statistical tests applied according to distribution: t-test for normally distributed continuous variables and Mann–Whitney U test for non-normal continuous variables. Statistical significance was considered at p < 0.05. PPF: periprosthetic fracture.

Variable	PPF (n = 15)	No PPF (n = 312)	Test used	p-value
Operative time (min)	145 (130–165)	120 (100–135)	Mann–Whitney U test	0.026
Blood loss (mL)	480 ± 120	360 ± 90	t-test	0.011

## Discussion

This multicenter retrospective study evaluated the incidence, risk factors, and outcomes of PPFs following total hip and knee arthroplasty. The overall incidence of PPF was 4.6%, slightly higher in hip arthroplasty (5.8%) compared to knee arthroplasty (3.2%). Older age, osteoporosis, and revision procedures were identified as independent risk factors for PPF, while female gender showed a trend toward significance. Rheumatoid arthritis and corticosteroid use were not present in the study cohort and thus did not influence the analysis. Surgical management included ORIF and revision arthroplasty, with postoperative complications occurring in 33.3% of fracture cases, including infection, nonunion, and re-operation. Operative complexity, reflected by longer operative time and higher blood loss, appeared associated with increased fracture risk. The short-term mortality in this study was low (6.7%), but functional and clinical outcomes were significantly impacted by fractures. When compared to existing literature, the incidence of PPF in this study (4.6% overall, 5.8% in THA, and 3.2% in TKA) falls within reported ranges, though slightly higher than some series [23]. This may be explained by the inclusion of both primary and revision procedures, as revision surgery is a known risk factor [24]. Advanced age and osteoporosis also emerged as significant predictors, consistent with prior reports linking reduced bone quality to higher fracture risk [25]. Revision procedures further increased incidence, supporting evidence that altered biomechanics and surgical history predispose to fractures [26]. The distribution of fracture types also mirrored published literature. In hips, Vancouver B1 and B2 fractures were most frequent, while Su type I predominated in knees [27]. Postoperative complications, including infection, nonunion, and re-operation, were comparable with other studies [28]. Despite advances in fixation and implant design, PPFs remain surgically challenging. Overall, these findings align with global evidence while emphasizing the importance of optimizing bone health, surgical technique, and perioperative care to improve outcomes [29].

Limitations

Despite the study’s strengths, including multicenter design and comprehensive evaluation of hip and knee arthroplasty patients, several limitations exist. The retrospective design limits causal inference and may cause selection bias. Insufficient fracture occurrences diminish the statistical ability to identify correlations with less prevalent risk factors. Radiographic assessment and fracture classification relied on available medical records, which may introduce misclassification bias. The study was also limited by the lack of routine assessment of BTMs and incomplete data on serum vitamin D and calcium levels, which may have influenced fracture risk assessment. Additionally, rheumatoid arthritis and corticosteroid use were not present in the study cohort, limiting the generalizability of findings to these patient populations. Future studies should focus on prospective multicenter designs with larger patient cohorts to validate risk factor associations and improve fracture prediction models. Investigations into preventive strategies, including bone quality optimization, implant selection, and surgical technique refinement, are warranted. Long-term follow-up to assess functional outcomes, implant survival, and quality of life after PPF management is also recommended to inform evidence-based care pathways.

## Conclusions

According to this multicenter study, PPFs after total hip and knee replacements are a serious complication, occurring in 4.6% of patients, with a higher incidence following hip arthroplasty (5.8%) compared to knee arthroplasty (3.2%). Advanced age, osteoporosis, and revision procedures were independent risk factors, while surgical complexity contributed to fracture occurrence and postoperative complications. Most fractures required surgical management, and outcomes were frequently affected by nonunion, re-operation, or infection. The study was limited by a lack of assessment of BTMs, vitamin D, and calcium levels, and the absence of rheumatoid arthritis and corticosteroid use in the cohort. These findings emphasize the necessity of thorough preoperative evaluation, bone health optimization, and careful surgical planning to reduce fracture risk.
